# Novel Algorithm of Network Calcium Dynamics Analysis for Studying the Role of Astrocytes in Neuronal Activity in Alzheimer’s Disease Models

**DOI:** 10.3390/ijms232415928

**Published:** 2022-12-14

**Authors:** Elena V. Mitroshina, Alexander M. Pakhomov, Mikhail I. Krivonosov, Roman S. Yarkov, Maria S. Gavrish, Alexey V. Shkirin, Mikhail V. Ivanchenko, Maria V. Vedunova

**Affiliations:** 1 Department of Neurotechnology, Institute of Biology and Biomedicine, Lobachevsky State University of Nizhny Novgorod, Nizhny Novgorod 603022, Russia; 2 Institute of Applied Physics RAS, 46 Ulyanov Street, Nizhny Novgorod 603950, Russia; 3 Department of Applied Mathematics, Lobachevsky State University of Nizhny Novgorod, Nizhny Novgorod 603022, Russia; 4 Prokhorov General Physics Institute of the Russian Academy of Sciences, Vavilova St. 38, Moscow 119991, Russia; 5 Laser Physics Department, National Research Nuclear University MEPhI, Kashirskoe Sh. 31, Moscow 115409, Russia

**Keywords:** astrocytes, neuron–astroglia networks, Alzheimer’s disease, β-amyloid, calcium activity

## Abstract

Accumulated experimental data strongly suggest that astrocytes play an important role in the pathogenesis of neurodegeneration, including Alzheimer’s disease (AD). The effect of astrocytes on the calcium activity of neuron–astroglia networks in AD modelling was the object of the present study. We have expanded and improved our approach’s capabilities to analyze calcium activity. We have developed a novel algorithm to construct dynamic directed graphs of both astrocytic and neuronal networks. The proposed algorithm allows us not only to identify functional relationships between cells and determine the presence of network activity, but also to characterize the spread of the calcium signal from cell to cell. Our study showed that Alzheimer’s astrocytes can change the functional pattern of the calcium activity of healthy nerve cells. When healthy nerve cells were cocultivated with astrocytes treated with Aβ42, activation of calcium signaling was found. When healthy nerve cells were cocultivated with 5xFAD astrocytes, inhibition of calcium signaling was observed. In this regard, it seems relevant to further study astrocytic–neuronal interactions as an important factor in the regulation of the functional activity of brain cells during neurodegenerative processes. The approach to the analysis of streaming imaging data developed by the authors is a promising tool for studying the collective calcium dynamics of nerve cells.

## 1. Introduction

Currently, calcium imaging is one of the most widely used methods for studying intercellular interactions in the central nervous system at the systemic level. However, researchers face methodological difficulties when studying intracellular functional calcium dynamics. These difficulties are related to the complexity of multidimensional data analysis obtained from many cells in the neuron–astroglia network. Therefore, novel approaches for identifying interactions between cells in the neuron–astroglia system and revealing features of the functioning of individual cells, both in physiological and complex pathological processes modelling, are of particular interest. With the increase in life expectancy, the incidence of Alzheimer’s disease (AD) has increased annually [1,2], which is why the study of the pathogenetic mechanisms of AD and the search for new targets for its therapy are among the most relevant topics in neurobiology and medicine. The features of the long-term presymptomatic development of AD pathological processes, its polyetiological nature, and the lack of effective strategies to prevent neurodegeneration are the reasons for close attention to this field all over the world [3,4].

The main distinguishing histological/biochemical features of AD are the abnormal accumulation of amyloid beta (Aβ) protein, in the form of extracellular plaques in the brain parenchyma and capillaries, and abnormal tau protein phosphorylation, which forms neurofibrillary tangles [5]. The accumulation and aggregation of Aβ and phosphorylated tau occurs gradually: monomers combine into oligomers in neurons and then assemble into fibrils, leading to the formation of amyloid plaques and neurofibrillary tangles [6,7]. It should be noted that the age of AD manifestation varies greatly. In recent years, the number of cases of early manifestation of clinical signs of AD has increased [8].

There is an increasing amount of information on the role of glial cells, and astrocytes in particular, in the pathogenesis of AD [9]. Astrocytes not only perform a structural and trophic function, maintaining the energy balance of neurons, which is extremely important in neurodegenerative processes, but also regulate synaptic transmission, participating in synaptic plasticity. Thus, astrocytes are active signaling partners of neurons. Astrocytic signaling and calcium activity are critical for many important physiological processes including metabolism, neurotransmitter clearance from the synaptic cleft, and integrated brain functions [10,11].

Changes in astrocyte function have been observed in the brains of people with AD, as well as through in vitro and in vivo models of AD. It is known that, in AD, astrocytes become reactive, which is characterized by an increased expression of intermediate filament proteins and cellular hypertrophy, as well as the production of inflammatory cytokines (IL-1, IL-6, and TNF-α) [9]. Reactive astrocytes are in close association with amyloid beta (Aβ) deposits. Synaptic transmission and the functioning of neural networks in the development of neurodegenerative processes can be directly modulated by reactive astrocytes, potentially contributing to cognitive decline in AD [12,13,14]. Moreover, recent studies directly linked astrocytes to the pathophysiology of AD. It is assumed that the reaction of astrocytes to β-amyloid plaques and neurofibrillary tangles leads to a loss of their neurotrophic potential and an increase in neurotoxic properties [15].

One of the most important mechanisms of interastrocytic signaling is the generation of calcium waves. Calcium signals allow astrocytes to interact with each other and function as a functional syncytium or astrocytic network [10]. Calcium events regulate the release of gliotransmitters, allowing astrocytes to modulate synaptic transmission and many other intracellular processes [12]. The dysregulation of Ca^2+^ signaling is widely regarded as an important component of neurodegenerative disease in general and Alzheimer’s disease in particular [16].

In 1994, Z.S. Khachaturian formulated the Ca^2+^ hypothesis of AD pathogenesis based on the similarity between the processes that occur in neurons during aging and AD [17]. Many physiological processes mediated by Ca^2+^ can pass into a pathological cascade during aging and AD. For example, senescent neurons experience an increased Ca^2+^ influx during the depolarization phase, which increases the resting Ca^2+^ concentration ([Ca^2+^]i), causing excitotoxicity. The acute impairment of Ca^2+^ homeostasis and Ca^2+^ signaling in the presence of β-amyloid was confirmed experimentally. A treatment with β-amyloid (at concentrations ranging from 100 nM to 5 μM) for several hours increased Ca^2+^ levels in the resting state by two to three times above the initial levels [12].

It is known that a dysfunction of endoplasmic reticulum (ER)-mediated Ca^2+^ signaling develops in the familial form of AD. I.B. Besprozvannyi’s group showed that presenilins can function as passive channels for the leakage of Ca^2+^ from the endoplasmic reticulum (ER). Mutations in the *PSEN1* gene cause disturbances in the stationary content of Ca^2+^ in the ER in the resting state and contribute to excessive accumulation of Ca^2+^ [18]. It was also shown that the expression of ryanodine receptors on neurons increases in various AD mouse models [19]. Treatment with dantrolene, an inhibitor of RyRs, normalizes impaired Ca^2+^ signaling and reduces Aβ deposition [20].

To date, huge investments in the development of AD therapy methods, aimed at correcting neuronal homeostasis, have not brought significant results. In this regard, more and more attention is being paid to astroglia as a new target for the correction of neurodegenerative processes, since the morphology and functions of astroglia can be regulated using various pharmacological interventions [21,22]. In view of the above, the study of the features of the transmission of calcium signals by astrocytes and their influence on the functioning of neurons is a highly relevant issue.

Our study aimed to investigate the features of the collective dynamics of calcium activity in astrocytes in modeling β-amyloidosis as a key component of AD pathogenesis. Moreover, we evaluated network calcium activity when AD astrocytes were cocultivated with healthy nerve cells

## 2. Results

### 2.1. Development of Algorithms for the Analysis of Network Characteristics of Calcium Activity of Neuron–Astroglia Networks

We aimed to study the features of intercellular calcium signaling in AD modeling. To do this, the network responses of cells in culture should be assessed, and characteristics of calcium activity should be identified to indicate the connectivity and responses of astrocytes. Previously, we developed the AstroLab algorithm, published in [23]. This algorithm takes into account the temporal correlations of calcium events. A calcium event is characterized as a continuous period of time at which a calcium signal exceeds two exponential moving averages (EMA) of the signal. The average intensity value over all pixels within the corresponding spatial region was used as a calcium signal of the cell. A space–time filter for noise reduction (BM3D) was applied to the signal intensity beforehand.

Measuring the number of simultaneously occurring events allows us to evaluate the activity of cultures. In the present study, we propose an algorithm that considers the delay in the onset and maximum of calcium events to determine the outline of calcium signal propagation and construct a dynamic graph. This makes it possible to determine the signal propagation between individual cells. The construction of a directed graph allows us to describe in more detail the network characteristics of calcium activity, as well as to identify signal transmission between astrocytes and determine “signal concentrators”—hubs.

#### 2.1.1. Algorithm for Detecting Events and Searching for Their Boundaries

The most important task in analyzing nerve cell calcium activity is the relevant identification of significant calcium events. This was performed by detecting event maxima, determining the baseline of activity, and searching for event boundaries.

The algorithm for detecting event maxima was based on a two-step approach:

(1) Finding the average line. The average line characterizes the signal component that changes slowly with time. The behavior of the intensity signal is characterized by a deviation from the average line and then returning to it. The average line can be found using the formula of exponential moving average (EMA):

(1) $$EMA_{j}^{\left(F\right)}=EMA_{j-1}^{\left(F\right)}\cdot k+\left(1-k\right)\cdot I_{j},\;j=\bar{1,T},$$

where *I* is the intensity signal array, *T* is the number of video frames, and *k* is the filtration coefficient, taking values in the range (0, 1). Index F means the forward direction of calculation from the beginning of the signal. To calculate it, only its value at the previous moment of time is required, which can speed up calculations compared to sliding-window algorithms.

(2) Searching for the maxima of intensity line sections that are above the average line:

(2) $$t=\left\{j,EMA_{j}^{\left(F\right)}=I_{j}\right\}\;,\;j=\bar{1,T},\;I^{m}=\left\{\max(I_{j}),t_{k}\leq j\leq t_{k+1}\right\},\;k=\bar{1,\left|t\right|}$$

To estimate the amplitude of the calcium activity event, it is necessary to calculate a baseline from which the measurement is carried out.

To find the baseline of a signal, the following steps are necessary:

- Find the local minima of the intensity line (minima of the sections of the intensity line that are under the middle line);
- Perform piecewise-linear interpolation of sections between the discovered local minima.

(3) $$BL_{j}=I_{k}^{m}+\frac{\left(j-t_{k}\right)\cdot \left(I_{k}^{m}-I_{k+1}^{m}\right)}{t_{k+1}-t_{k}},j=t_{k}\ldots t_{k+1},$$

where $t_{k},I_{k}^{m}$ are the time and intensity of the minimum.

**Figure 1:**
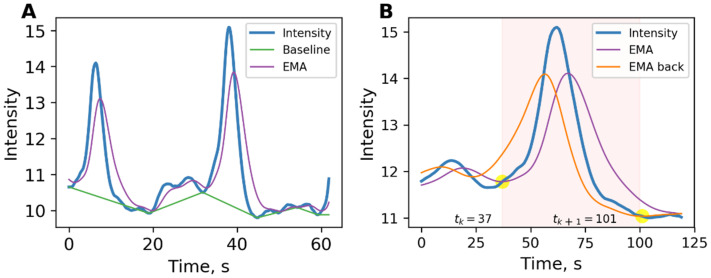
(**A**) Intensity signal shown in blue. EMA (exponential moving average) is used to find the baseline. (**B**) Method of searching for the start and finish of an event. The moment when the intensity signal (shown in blue) crosses the violet EMA line is taken as the start of the event. The moment when the intensity signal crosses the orange EMA back line is taken as the finish of the event. Event time highlighted in pink.

Figure 1A shows the intensity signal, mean, and baseline.

Each event is a period defined by its start and end times. The exact determination of the boundaries of events is important for the operation of the algorithm for constructing a dynamic graph. The EMA lines were applied to find the event boundaries, as shown in Figure 1B. One of them has already been calculated during the search for local maxima, so fewer calculations are required. The event’s start was determined as the point when the intensity line crossed the exponential moving average calculated according to (1). The finish of the event was determined as the moment the intensity line crossed the exponential moving average calculated from the finish of signal in accordance with the following:

(4) $$EMA_{j}^{\left(B\right)}=EMA_{j+1}^{\left(B\right)}\cdot k+\left(1-k\right)\cdot I_{j},\;j=\bar{1,T},$$

where *k* is the filtration coefficient that takes values in the range (0, 1), and index B is the reverse direction of calculation from the finish of the signal.

An event was considered significant if the deviation of the maximum event point from the baseline exceeds a given threshold. A threshold that skips the events of spontaneous calcium activity, typical for a monoastrocytic culture and more significant events, was chosen.

Thus, a set of events was obtained as follows:

(5) $$Ev^{i}=\left\{\left(start,finish\right):EMA_{start}^{\left(F\right)}=I_{start};EMA_{finish}^{\left(B\right)}=I_{finish};start\leq finish\right\},\;i=\bar{1,n}$$

To tune the algorithm, searching for the optimal *EMA* filtration coefficient is important. For this purpose, we marked the start and finish of events of the given cell intensity of one neuronal culture in the marking program.

It is required to minimize the total discrepancy between the starts and finishes of the algorithmic markup from the manually performed markup:

(6) $$mark_{err}=\overset{\left|Ev\right|}{\underset{i=1}{\sum }}\left(\left|start_{i}^{m}-start_{i}^{a}\right|+\left|finish_{i}^{m}-finish_{i}^{a}\right|\right)$$

where $start_{i}^{m}$ represents the start of the calcium activity event when marking manually; $start_{i}^{a}$ is the start of the calcium activity event as a result of algorithmic markup; $finish_{i}^{m}$ is the end of the calcium activity event when marking manually; and $finish_{i}^{a}$ is the end of the calcium activity event that resulted from the algorithmic markup.

**Figure 2:**
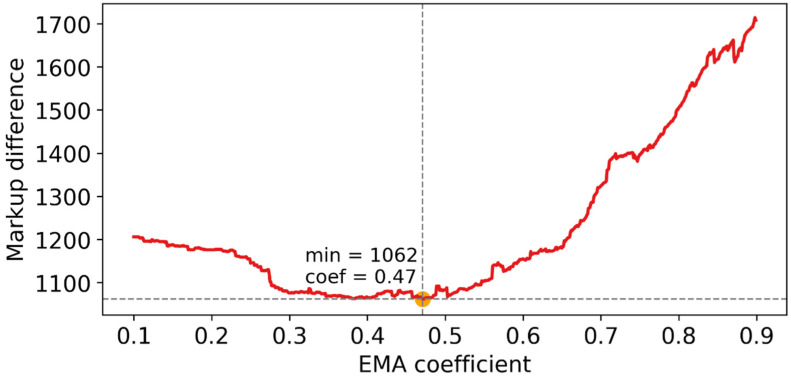
The figure shows how the total deviation of the event boundaries marked by the expert depends on the event boundaries found by the algorithm for different values of the EMA coefficient.

By changing the filtration coefficient, we were able to plot a graph of the markup mismatch (Figure 2).

#### 2.1.2. Construction of a Dynamic Calcium Signal Propagation Graph

Calcium signal propagation in neuron–astroglia networks can be described using a directed graph. Directed graph technology is often used in the search for the optimal route in navigation systems and data compression. In this graph, the vertices will correspond to cells, and the edges indicate calcium activity signaling between cells and their direction.

The calcium signal propagation graph was constructed by comparing the overlay of events identified on the intensity time series of one region with the intensity time series events of the regions adjacent to it.

The algorithm for constructing a dynamic graph was based on the analysis of the overlay of events and was implemented in the MATLAB environment.

For each frame, a directed graph can be constructed as follows:

(7) $$G_{t}=\langle V_{t},E_{t}\rangle ,\;t=\bar{1,T}$$

An edge is drawn between two vertices if the following conditions are satisfied simultaneously:

1. Intersection of time intervals corresponding to events.

(8) $$C\left(ev_{p}^{i},ev_{q}^{j}\right)=[start_{p}^{i}<start_{p}^{j}<finish_{q}^{i}<finish_{p}^{j}]$$

2. Let us define the set of vertices of a directed dynamic graph as follows:

(9) $$V_{t}=\left\{v_{i}^{t},:\;i\;cell\;at\;monent\;t,i=\bar{1,n}\right\}$$

(10) $$E_{t}=\left\{e_{ij}=\left(v_{i},v_{j},≺\right):v_{i},v_{j}\in V_{t},ev_{p}^{i}\in Ev^{i},ev_{q}^{j}\in Ev^{j},C\left(ev_{p}^{i},ev_{q}^{j}\right)\wedge \left(a_{ij}=1\right)\right\}$$

Their totality is a directed dynamic graph for the entire video sequence:

(11) $$G_{D}=\langle V_{D}\rangle ,E_{D}$$

The set of vertices of a dynamic graph is the union of the vertices $V_{t}$:

(12) $$V_{D}=\underset{t}{\cup }V_{t}$$

The set of edges of a dynamic graph is the union of the edges $E_{t}$:

(13) $$E_{D}=\underset{t}{\cup }E_{t}$$

**Figure 3:**
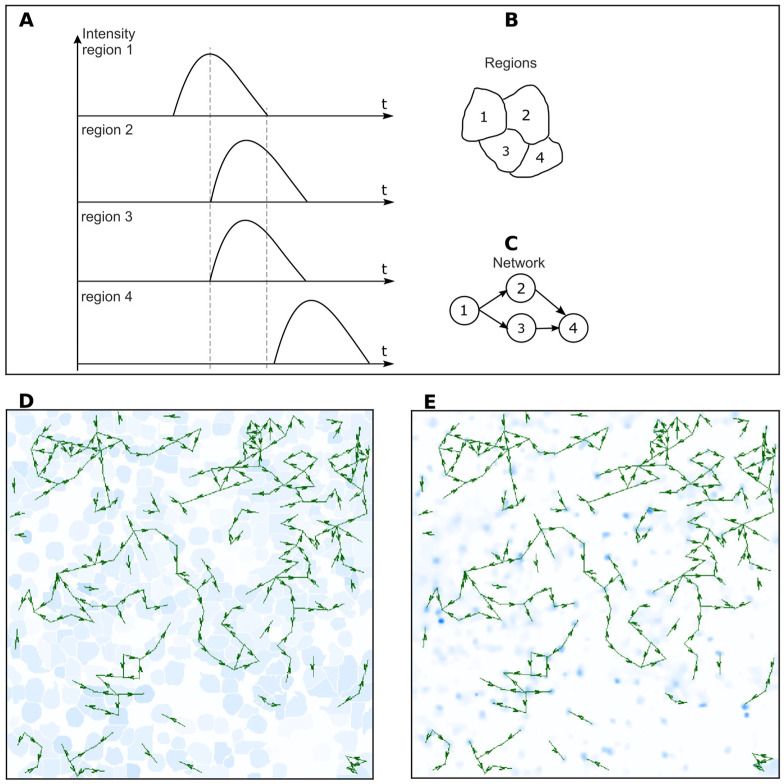
(**A**) Diagrams of intensity signals in four adjacent regions. (**B**) Mutual arrangement of these regions. (**C**) Resulting graph. The signal propagates from cell 1 to cells 2 and 3, and from cell 3 to cell 4. (**D**) An example of a video sequence frame formed during the visualization of the resulting dynamic graph. (**E**) The noise-filtered original video sequence obtained from a microscope with a light blue color map.

An example of a dynamic graph construction is shown in Figure 3.

The expected assumption is that the signal propagates from the region where it appears earlier. In Figure 3A, the calcium event in region 1 started earlier than in regions 2 and 3, and the event in region 4 lagged behind the event in region 3.

Thus, the resulting directed signal propagation graph is shown in Figure 3C; the signal propagated from cell 1 to cells 2 and 3, and from cell 3 to cell 4.

#### 2.1.3. Implementation of the Dynamic Graph Construction Algorithm

An array that stores a dynamic graph is a matrix of size *n* × *n* × *m*, where *n* is the number of cells and *m* is the number of frames in the source video. Each time slice of the array represents the adjacency matrix of the dynamic graph for the current frame.

Two outer-nested loops enumerated all pairs of cells. All events on their intensity time series were compared for a pair of neighboring cells. This is performed using two internal loops in which the overlaps of all calcium activity events that occurred in these cells are compared. If both events are significant and overlap in time, a new edge is marked in the adjacency matrices during the entire period of event overlap. Depending on which event starts earlier, the direction of the edge is determined.

The resulting dynamic graph is visualized by recording a video file. After analyzing the interactions of all pairs of cells, a frame-by-frame output of the video sequence is carried out. The video sequpience is formed from two components: The image of the substrate, which is a processed original image, on which the arrows of the graph edges denoting intercellular connections are superimposed. Two variants of the substrate image are formed while recording the resulting video sequence. The first option is shown in the video frame in Figure 3D. Here, each region is filled with one color, showing the deviation of the average intensity of the region from the baseline. The second version, shown in Figure 3E, uses the noise-filtered original video sequence obtained from a microscope, constructed using the jet or light blue color map.

The arrows are drawn between the centers of the regions, which were calculated by averaging the coordinates of the elements of the array describing which region the points belonged to. The color of the arrows was chosen for reasons of contrast.

On the first variant of the substrate, it is convenient to observe the direction of calcium signal propagation; on the second one, one can compare the algorithm’s results with the actual signal propagation along the cell processes.

Examples of representative video of raw calcium activity are given in Supplementary Video S1 (neuronal culture) and Supplementary Video S2 (astrocytic culture). Examples of representative dynamic graphs in video format are given in Supplementary Video S3 (neuronal culture) and Supplementary Video S4 (astrocytic culture).

#### 2.1.4. Computing Dynamic Graph Metrics

Based on the generated dynamic graph, several metrics are calculated.

The number of edges in the dynamic graph represents the total number of edges across all frames.

(14) $$\left|E_{D}\right|=\overset{T}{\underset{t=1}{\sum }}\left|E_{t}\right|$$

This metric considers the edge on each frame for the overlapping time of the compared events.

The number and share of active cells: A cell was considered active if there was a transmission to a neighboring cell at least once, reception from a neighboring cell, or significant events in neighboring cells occurred simultaneously (the direction of transmission cannot be determined).

(15) $$nc=\left|V_{D}\right|$$

(16) $$pc=\frac{100\cdot \left|V_{D}\right|}{n}$$

The maximum number of transmissions per frame represents the number of transmission events between adjacent cells for the video frame on which their maximum number is registered.

(17) $$\underset{t}{\max}\;\left|E_{t}\right|,\;\;E_{t}\in E_{D}$$

The average number of periodic connections per cell. This indicator does not take into account those links that appear only once in the entire video.

(18) $$\frac{\sum_{v_{i}\in V_{D}}\left({deg}^{-}(v_{i}\right)+{deg}^{+}(v_{i}))}{n}$$

Next, the graph is “compressed” in time, which results in a weighted directed graph that characterizes the number of registered transmissions between each pair of cells, and its metrics are calculated.

A weighted directed graph is a pair $W=\left(G,\;B\right)$, where $G=\left(V,\;E\right)$ is an ordinary directed graph, and B is a weight function. The weight function of a weighted directed graph can be given by a matrix B, whose element $b_{ij}$ equals the value of the weight function on the arc $\left(i,\;j\right)$ if the arc leads from vertex i to vertex j, or is otherwise zero. This matrix will be called the arc label matrix.

The vertices of a weighted directed graph correspond to cells.

(19) $$V_{C}=\left\{v_{i},:i-\mathrm{th}\;\mathrm{cell},i=\bar{1,n}\right\}$$

The arc label matrix is constructed as follows:

(20) $$b_{ij}=\underset{e_{ij}\in E_{t},E_{t}\in E_{D}}{\sum }\left[e_{ijt}=≺\;\wedge e_{ijt-1}\neq ≺\right]-\;-\underset{e_{ij}\in E_{t},E_{t}\in E_{D}}{\sum }\left[e_{ijt}=≻\;\wedge e_{ijt-1}\neq ≻\right]\;i=\bar{1,n},\;\;j=\bar{1,n},\;\;t=\bar{1,T}$$

Its set of edges is calculated as follows:

(21) $$E_{C}=\left\{e=\left(v_{i},v_{j},≺\right):v_{i},v_{j}\in V_{C},b_{ij}>0\right\}\cup \left\{e=\left(v_{i},v_{j},≻\right):v_{i},v_{j}\in V_{C},b_{ij}<0\right\}$$

An important parameter needed to characterize network activity is the number of edges on a compressed graph. Unlike the number of edges in a dynamic graph, in a compressed graph an edge is counted only once for each transmission.

(22) $$N_{EC}=\left|E_{C}\right|$$

A low parameter value may indicate the death of cells or a violation of their correct functioning.

**Figure 4:**
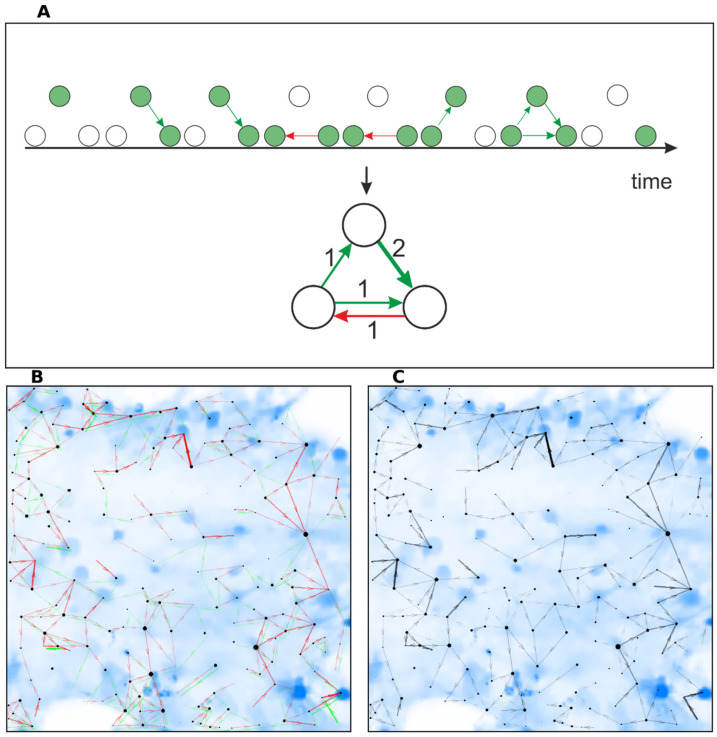
(**A**) Principle of transition to a compressed graph. Example for three adjacent cells for eight consecutive frames. Green and red colors of directed links encoding the direct and reverse transmission between cells (circles). Active cells are shown by the green color filling. Finally, the compressed graph is constructed by counting of directed links (transmissions). Transmission in one direction within several frames is counted only once. The number of transmissions in each direction is counted separately. (**B**) Visualization of a compressed graph. Transmissions in both directions are shown with arrows of different colors (red and green). (**C**) Visualization of a compressed graph. The line contrast increases in proportion to the difference between incoming and outgoing transmissions with arrow in the prevailing direction. The size of the vertices is proportional to the indegree metric, describing the number of incoming edges.

The metrics of centrality or proximity to the center determine the most important cells, which in graph theory and network analysis are the graph’s vertices. The centrality metrics hubs and authority are two related recursive measures (Figure 4).

The centrality metrics indegree and outdegree are based on the number of edges connecting to each node. Outdegree is the number of transmissions outgoing from each cell. Indegree is the number of transmissions received by the cell.

#### 2.1.5. Construction of Group Histograms

A method for constructing group histograms was implemented to analyze the activity of cells from several cultures belonging to the same experimental group. Different cultures in a group may have different types of activity, and a group histogram can be a tool that summarizes the activity characteristics of cells of different cultures in a group, forming a group portrait. As the number of cells is different for different cultures, and groups can have different numbers of cultures, the indicator is normalized per hundred cells to ensure the possibility of comparing different groups.

The number of active cells per hundred with *i* transfers for all cultures in the group:

(23) $$n_{i}=\frac{100}{n_{g}}\cdot \overset{K}{\underset{k=1}{\sum }}\overset{n_{k}}{\underset{j=1}{\sum }}\left[\left({deg}^{-}(v_{j}^{k}\right)+{deg}^{+}(v_{j}^{k}))=i\right]\;,v_{j}^{k}\in V_{D},\;i=\bar{1,\;50},$$

$n_{k}$—number of cells in culture;

*K*—number of cultures in the group;

${deg}^{-}(v)$—number of edges for which v is the initial vertex (out-degree);

*n_g_*—number of cells in all cultures.

(24) $$n_{g}=\overset{K}{\underset{k=1}{\sum }}n_{k}$$

A necessary step in our work was to confirm the applicability of the algorithm we developed not only on astrocytic cultures, but also on mixed cultures containing neurons. The algorithm of calcium activity analysis we developed is most suitable for the analysis of astrocytic cultures, since the current implementation of the method involves the analysis of connections of neighboring cells. It is known that calcium ions are transferred between astrocytes either via gap junctions or purinergic transmission, where ATP is the mediator. ATP binds to the P2 receptor and triggers a cascade that releases calcium from intracellular depots. This type of transmission range is only about 17 μM [16,24]. In addition, calcium waves can trigger mGluR receptors in response to an increase in glutamate concentration at nearby synaptic junctions. This makes it reasonable to consider signal transmission directly between neighboring cells in astrocytic cultures.

For cultures containing neurons, which can contact each other through neuronal processes over significant distances, an algorithm for analyzing connections not only between neighboring but between all culture cells looked promising. The tests on astrocytic cultures revealed that such an algorithm found false long-range connections. In this regard, searching for a universal method for comparing neuronal cultures with astrocytic ones is interesting.

It was decided to focus on an algorithm that analyzes only short-range connections as a universal algorithm at this stage. This choice was based on the following postulates: Hippocampal cell cultures include astrocytes, and such cultures have transmissions between the adjacent neurons. As the total activity of the culture increases, so does the activity in terms of transmissions between neighboring cells. The application of the algorithm will make it possible to correctly analyze the activity of mixed cultures and avoid errors when analyzing monoastrocytic cultures.

The developed algorithm pipeline is presented in Supplementary Figure S1.

To test this assumption, we compared the activity parameters of intact primary hippocampal cell cultures containing both neurons and astrocytes with primary monoastrocytic cultures when analyzed using the developed algorithm.

**Figure 5:**
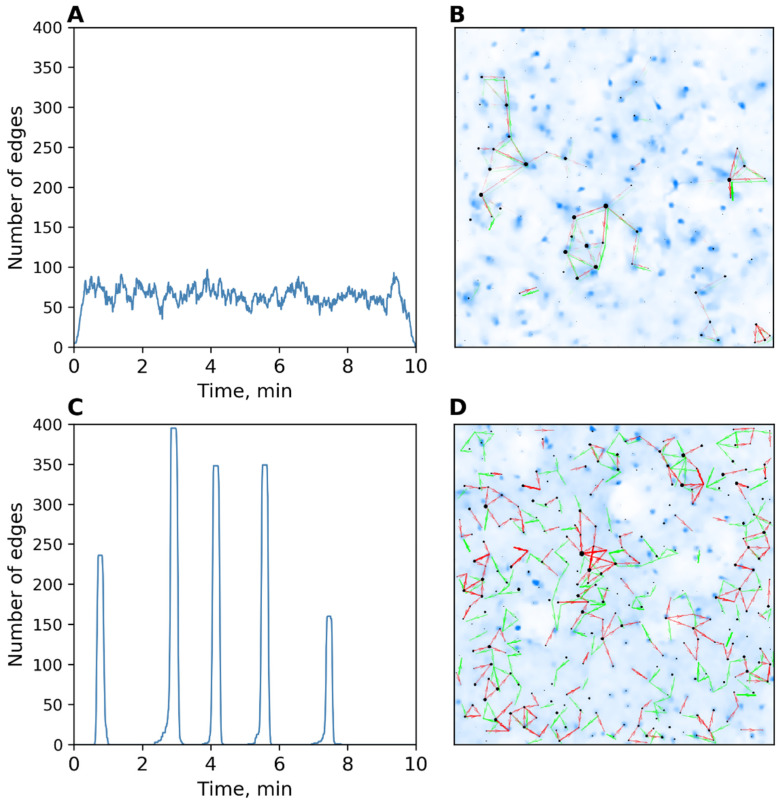
(**A**) Dynamics of the number of transmissions in an intact astrocytic culture. (**B**) Map showing the uniformity of transmissions in an intact astrocytic culture. (**C**) Dynamics of the number of transmissions in an intact neuronal culture. (**D**) Map showing the uniformity of transmissions in an intact neuronal culture.

Intact hippocampal cell cultures (“neuronal cultures”) were shown to be characterized as having high synchrony and uniform calcium signaling (Figure 5A,B). Figure 5C,D show the dynamics of the number of transmissions of a monoastrocytic culture. It is important to note that no synchronization or uniformity of spontaneous activity were observed in such cultures.

To compare the groups, we applied the method of group histogram. The results were obtained using the algorithm for finding events and their boundaries described in Section 2.1.1. The construction of the dynamic graph of calcium signal propagation and calculation of the number of its edges was carried out using the algorithm described in Section 2.1.2 and Section 2.1.3.

**Figure 6:**
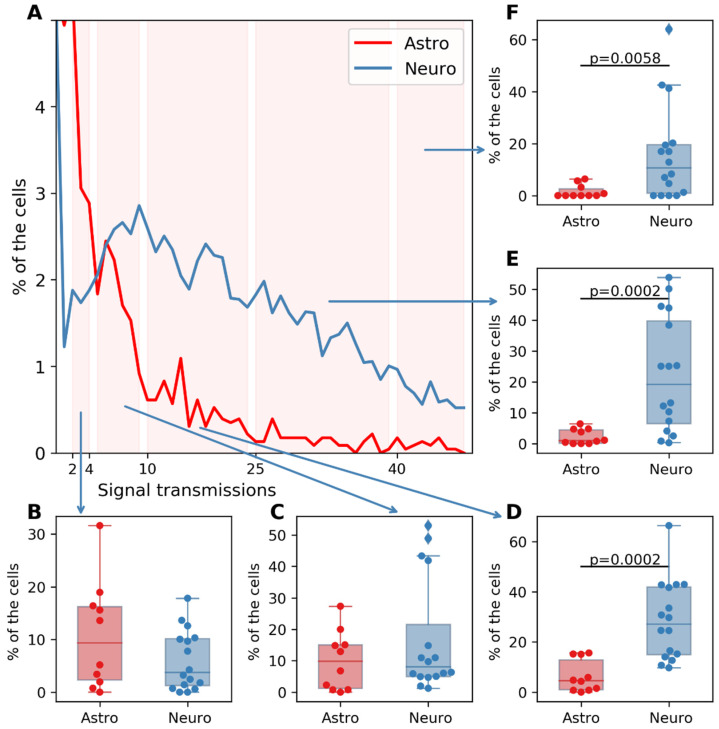
(**A**) Group histograms of cultures of intact astrocytes (red) and intact neurons (blue). (**B**) Percentage of cells with 2 to 4 transmissions. (**C**) Percentage of cells with 5 to 10 transmissions. (**D**) Percentage of cells with 11 to 24 transmissions. (**E**) Percentage of cells with 25 to 40 transmissions. (**F**) Percentage of cells with more than 40 transmissions. Significant differences between groups were assessed using Mann–Whitney–Wilcoxon tests.

Figure 6A represents the resulting group of histograms of cortical monoastrocytic cultures and primary hippocampal cell cultures containing neurons and astrocytes. The vertical axis shows the proportion of cells in which a certain number of transmissions were recorded. For statistical evaluation, the horizontal axis was divided into several intervals, in each of which the proportion of cells with the following number of calcium signal transmissions was calculated: 2 to 4, 5 to 10, 11 to 24, 25 to 40, and more than 40 transmissions. The data are presented in Figure 6B–F.

It was shown that in mixed neuron–astrocytic cultures, the proportion of cells in which more than five transmissions of calcium events was recorded was significantly higher. This coincided with data from the literature [25] regarding the number of calcium activity events for neuronal and astrocytic cultures, which confirmed our hypothesis about the applicability of the developed algorithm.

### 2.2. Study of Astrocyte Calcium Activity in the Modeling of Alzheimer’s Disease

In the second stage of our work, we studied the features of the calcium activity of astrocyte cultures in two experimental AD models. In a pharmacological model, chronic amyloidosis as the main pathogenetic component of the sporadic form of AD was modeled using synthetic fibrillar ß-amyloid peptide 1-42 (InnovaGen, Lund, Sweden). The second model of Alzheimer’s disease was the primary culture of astrocytes obtained from transgenic 5xFAD mice. These animals had mutations in the APP and presenilin transgenes that are typical for hereditary (familial) forms of Alzheimer’s disease.

**Figure 7:**
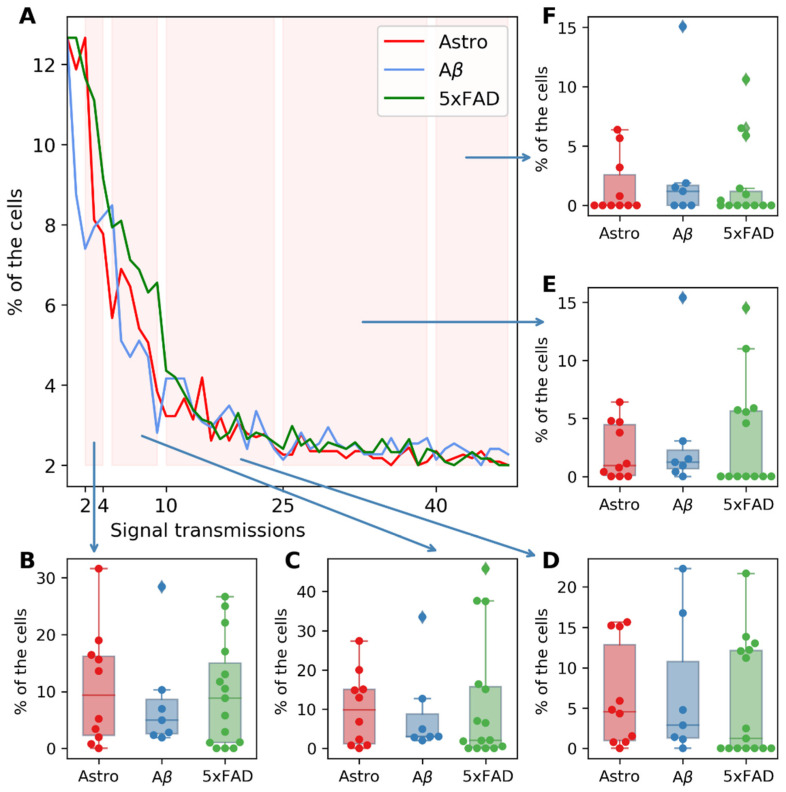
(**A**) Group histograms of cultures of intact astrocytes (red), astrocytes in the pharmacological modeling of AD (blue), and astrocytes obtained from transgenic animals (green). (**B**) Percentage of cells with 2 to 4 transmissions. (**C**) Percentage of cells with 5 to 10 transmissions. (**D**) Percentage of cells with 11 to 24 transmissions. (**E**) Percentage of cells with 25 to 40 transmissions. (**F**) Percentage of cells with more than 40 transmissions.

To assess the level of calcium activity, we investigated the number of calcium events transmitted between neighboring cells. For this purpose, group histograms of astrocyte cultures were built, which are shown in Figure 7A. It was demonstrated that the number of calcium events transmitted between cells, as well as the distribution of cells by the number of transmissions in all selected intervals, did not change compared to the control group in both studied AD models. Thus, no significant changes in the collective calcium dynamics of astrocytes treated with AB42, as well as in the transgenic model of 5xFAD astrocytes, were revealed.

### 2.3. Study of the Effect of Astrocytes with Experimentally Induced AD on the Functional Activity of Normal Nerve Cells

The brain neural networks’ interaction with astrocytes is fundamental for their correct operation: the astrocytes carry out the subtle regulation of neuronal activity by means of calcium activity and the release of gliotransmitters, neurotrophic factors, and a wide range of other bioactive substances. Therefore, in the third stage of our work, we studied the effect of altered astrocytes on the calcium activity of healthy neurons during their cocultivation. To date, no such studies have been carried out. However, several studies [26,27] have shown that cocultivating aging astrocytes with hippocampal neurons suppresses synaptic maturation and synaptic transmission. Our study sheds light on the effect of astrocytes in β-amyloidosis on the calcium activity of neuronal cells. The methods we developed for analyzing calcium imaging data are subtle and sensitive enough to analyze the activity of both neurons and astrocytes. After 14 days of AD modeling, healthy hippocampal cells were replanted into monoastrocytic cultures. There was no further application of β-amyloid to the “Aβ” and “Aβ + Neuro” groups. The calcium activity of monoastrocytic cultures and astrocytes cocultivated with healthy neurons was performed 14 days after the replantation of healthy cells. The calcium activity characteristics of 15,121 cells were processed.

**Figure 8:**
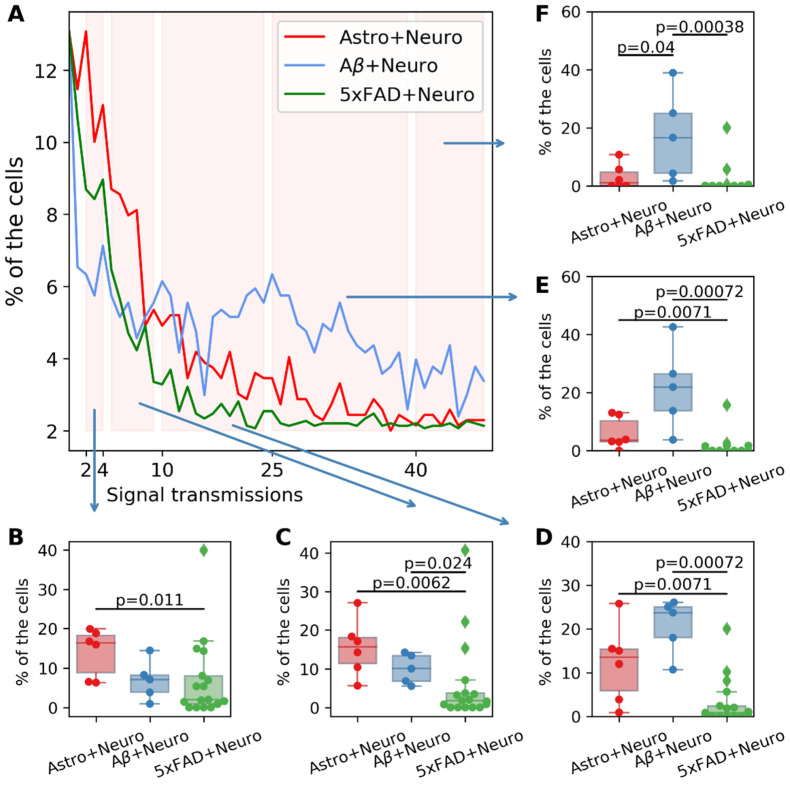
(**A**) Group histograms of cultures of intact astrocytes with replantation of healthy nerve cells (red), astrocytes in pharmacological modeling of AD with replantation of healthy nerve cells (blue), and astrocytes obtained from 5xFAD mice with replantation of healthy nerve cells (green). (**B**) Percentage of cells with 2 to 4 transmissions. (**C**) Percentage of cells with 5 to 10 transmissions. (**D**) Percentage of cells with 11 to 24 transmissions. (**E**) Percentage of cells with 25 to 40 transmissions. (**F**) Percentage of cells with more than 40 transmissions. Significant differences between groups were assessed using Mann–Whitney–Wilcoxon tests.

Figure 8 shows group histograms of the number of calcium signaling transmissions in cultures of intact astrocytes cocultivated with neurons compared to neurons cocultivated with astrocytes chronically exposed to β-amyloid and astrocytes derived from 5xFAD mice.

It was shown that modeling of β-amyloidosis led to an increase in network calcium activity in cocultivated nerve cells. In the “Aβ + Neuro” group, the share of cells with a high (10–50) number of transmissions of calcium events was significantly (*p* = 0.04) increased (in the “Astro+Neuro” control cultures, 25–40 events per 20 min were recorded in 1.2 [0.2; 4.9]% of cells, in the “Aβ + Neuro” group (cocultivation of neurons with astrocytes exposed to β-amyloidosis), 25–40 events per 20 min were recorded in 17.1 [5.0; 24.81]% of cells). It is important to note that the altered astrocytes had a stimulating effect, since β-amyloid was not directly added to the cultures after the hippocampal cells were replanted.

At the same time, in the group of healthy hippocampal nerve cells cocultivated with astrocytes obtained from 5xFAD transgenic animals (“5xFAD + Neuro”), the collective calcium activity was significantly reduced. The most pronounced decrease was noted in the intervals of 5–10 and 11–24 signal transmissions per cell (in the “Astro + Neuro” group, 5–10 transmissions of events between adjacent cells in 20 min were recorded in 16.1 [12.4; 18.2] % of cells, 11–24 event transmissions were recorded in 14.75 [6.32; 15.6] % of cells; in the “5xFAD + Neuro” group, 5–10 event transmissions in 20 min were recorded in 2.8 [0.95; 5.2] % of cells, and 11–24 event transmission were recorded in 1.3 [0.20; 3.64] % of cells). No cells with more than 25 transmissions were found in the “5xFAD + Neuro” group.

**Figure 9:**
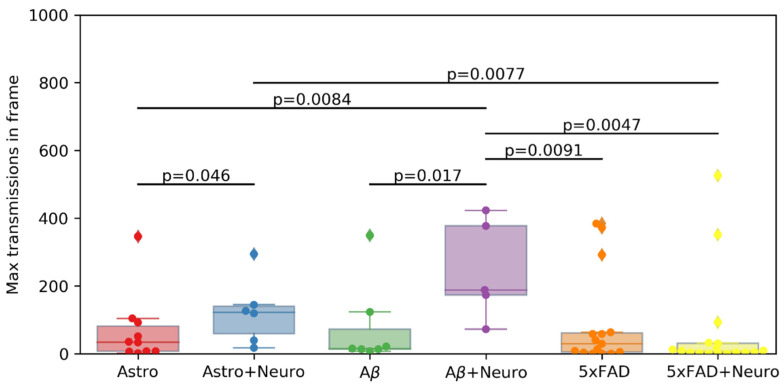
Comparison of the maximum number of calcium signal transmissions in a frame. The significance of differences between groups was assessed using a Mann–Whitney–Wilcoxon test.

The parameter of the maximum number of signal transmissions in a frame was also evaluated. The results of the pairwise comparison of samples for this parameter and the range diagram are shown in Figure 9. No significant differences were found between the experimental groups.

Thus, a study of the effect of astrocytes with experimentally induced AD on the functional activity of normal nerve cells using different models revealed opposite changes. The neuronal cell network calcium activity decreased when cocultivated with astrocytes obtained from newborn 5xFAD mice. At the same time, the cocultivation of hippocampal nerve cells with astrocytes previously exposed to recombinant fibrillar β-amyloid led to a hyperactivation of calcium activity.

To exclude the possibility that changes in calcium activity were associated with the death of some cells during AD modeling, we studied the viability of astrocyte cultures obtained from 5xFAD mice and astrocyte cultures after 14 days of β-amyloid application. For both cultures in the modeling of β-amyloidosis with recombinant amyloid 1-42 and the astrocyte cultures obtained from 5xFAD mice, cell viability was maintained at the level of control cultures and was 97–99%. Thus, changes in calcium activity were not associated with the triggering of cell-death programs and were of a different nature.

**Figure 10:**
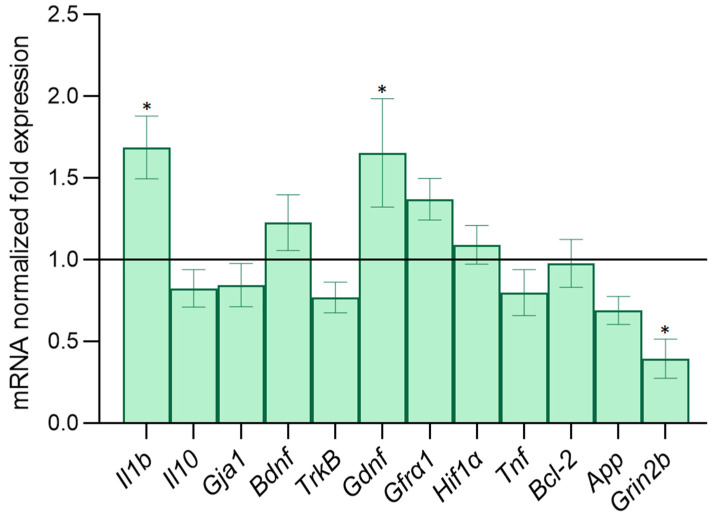
Level of mRNA expression of genes of interest in astrocytic cultures during chronic application of β-amyloid. Data are normalized relative to intact cultures. *—statistically significant difference between experimental groups, *p* ≤ 0.05 (one-way ANOVA and Tukey’s multiple post-hoc test).

It can be assumed that the effect of altered astrocytes on the activity of healthy cells was associated with the transition of astrocytes into a reactive state and a corresponding change in the spectrum of synthesized biologically active substances. These compounds primarily include proinflammatory cytokines and neurotrophic factors. Moreover, intercellular contacts, consisting of connexin proteins as well as glioreceptors, took part in the implementation of the collective calcium dynamics of astrocytes. Therefore, in the final stage of our work, we assessed the level of expression of a number of key regulatory molecules and receptors in astrocytic cultures against the background of the β-amyloid application. Using RT-PCR, we evaluated the mRNA expression of pro-inflammatory cytokines *Il1β* and *Tnf*; anti-inflammatory cytokine *Il10*; neurotrophic factors *Bdnf* and *Gdnf*, as well as their receptors; connexin 43, as one of the most important astrocytic connexins (*Gja1*) regulating the development of apoptosis protein *Bcl-2*; the hypoxia-induced *Hif1α* factor; and the expression of the GluN2B subunit of the NMDA glutamate receptor (*Grin2b*). The data are presented in Figure 10.

It was shown that the modeling of amyloidosis caused an increase in the expression of mRNA of the proinflammatory cytokine Il-1β, which indicated the activation of astrocytes. An increase in the neurotrophic factor GDNF mRNA expression due to astrocytes was also revealed, which can be regarded as an adaptive response of cells when exposed to a stress factor.

Interestingly, the expression level of mRNA encoding the GluN2B subunit of the NMDA receptor on astrocytes decreased, which may explain the effect of astrocytes altered in the modeling of chronic β-amyloidosis on neuronal activity when cocultivated with healthy hippocampal cells.

## 3. Discussion

One hypothesis for Alzheimer’s disease is called the calcium hypothesis. According to this, the activation of the amyloidogenic pathway and the formation of amyloid plaques affect neuronal Ca^2+^ homeostasis and lead to impairments in synaptic plasticity, learning, and memory. Aβ oligomers formed in the extracellular space can interact with the plasma membrane, causing hyperactivation of calcium channels (NMDAR, AMPAR, and VGCC). On the other hand, intracellular hyperphosphorylated tau protein can also contribute to the disruption of Ca^2+^ homeostasis in neurons [28,29,30]. As a result, an increase in the concentration of cytosolic Ca^2+^ leads to mitochondrial dysfunction and the subsequent development of ER stress and activation of apoptotic cell death. However, neuronal Ca^2+^ hyperactivity is known to be observed in mice even before the development of clinical symptoms of the disease and the formation of amyloid plaques. An increase in calcium oscillations in neuron cultures was also noted during the experimental modeling of AD [31]. In contrast, the calcium activity of neurons can decrease in the later stages of the disease and transform into hypoactivity [32,33,34]. In addition, some studies have reported that Aβ oligomers can impair synaptic activity by inhibiting P/Q-type calcium channels [35]. Thus, the question of the features of calcium dynamics in nerve cells in Alzheimer’s disease remains open, and the molecular–cellular mechanisms underlying it require careful study.

Accumulated experimental data strongly suggest that astrocytes play an important role in the pathogenesis of neurodegeneration and deserve detailed study [12,36]. Various pathological stimuli can induce morphological, molecular, and functional changes in astrocytes [35,36,37]. In addition to the long-described trophic and vasoregulatory functions, astrocytes directly contact synapses and participate in the regulation of synaptic plasticity. Astrocytes can generate calcium waves that are transmitted from cell to cell and act as intercellular signaling [24]. A variety of neurotransmitter receptors are expressed on the astrocytic membrane, affecting the intracellular level of Ca^2+^. Among these neurotransmitters are glutamate, GABA, norepinephrine, ATP [38], etc. The release of these neurotransmitters by neurons leads to an increase in intracellular Ca^2+^ levels in astrocytes through the release of Ca^2+^ from internal stores such as ER [10]. The increase in Ca^2+^ content propagates the cell processes, leading to a global increase in the Ca^2+^ content in the cytoplasm that can activate numerous astrocytes which are in contact with each other. On the other hand, the release of gliotransmitters can also regulate synapse function and calcium currents in neurons [10,24].

In view of this, astrocytes are an integral part of neural networks, since they dynamically interact with synapses, and mutually and bidirectionally regulate the activity of neurons, which reflects the concept of the “three-part synapse” [39]. Calcium waves generated by astrocytes modulate neuronal activity through cell-to-cell signal transmission [11]. To understand how changes in reactive astrocytes contribute to cognitive decline in AD, it is critical to study changes in neuron–glia interactions during AD pathogenesis, as they may directly affect neuronal function. It is important to note that, while much is currently known about disorders of neural network activity in AD, there are very little data on changes in the functional consolidation of astrocytic calcium activity and the effect of astrocytes on neuronal activity. The effect of astrocytes in Alzheimer’s disease on the calcium activity of neuron–astroglia networks was the object of the present study.

The study of patterns of calcium signal transmission between cells is a non-trivial task. The simplest way to identify patterns in the neural network is by arbitrarily choosing a subset of neurons and tracking their spike synchronicity [40]. Choosing the cells of interest by an expert provides the flexibility of pattern recognition, but it lacks impartiality, robustness, and reproducibility. The standard way to automate the process of finding similarities of calcium fluctuations between cells is to compute Pearson’s correlation coefficients between corresponding time series of the ROIs [41,42].

The further extension of this approach is using correlations between lagged signals and their derivatives, which provides the possibility to detect the cell transmitter and cell receiver in general [23]. However, real calcium events can have more complex transmission paths in cells and irregularly shift in time, which is a limitation of the method. Another way of assessing the synchronicity of fluctuations of the observed cells in the whole is computing a global synchronization index based on eigenvalues of the synchronization matrix [43], which indicates the synchronicity of collective cell fluctuations. In addition to the synchronization index, the eigenvalues can provide information about the number of synchronization clusters [44]. The automation of coactivated neuron detection can be achieved by computing the number of active neurons in the sliding window and identifying whether it is a significant number using statistical methods [43]. Previous investigations suggested using unsupervised clustering analysis to classify individual cells based on the properties of temporal activity patterns [45]. As an extension of those approaches, pairwise functional connections between cells can be represented as an oriented or nonoriented graph and can be studied using complex network analysis tools [42].

Previously, we proposed an algorithm for processing imaging data represented by an astrocytic network in the form of a directed graph, of which the vertices correspond to individual astrocytes and the edges indicate functional relationships between astrocytic events. The value of the correlation between the average levels of calcium in cells was used as a numerical characteristic to assess the connectivity of the astrocytic network. The functional relationship between cells was defined as the excess of the threshold by the level of correlation of the processed calcium signals of the cells, considering a signal delay of ±5 s. The average calcium level in a cell was defined as the average value of the intensity within the area corresponding to the cell for some point in time [23].

In the present study, we have expanded and improved our approach’s capabilities to analyze calcium activity. The approach proposed in this paper to construct dynamic directed graphs of both astrocytic and neuronal networks allowed us to not only identify functional relationships between astrocytes and determine the presence of network activity, but also to characterize the spread of the calcium signal from cell to cell. The new algorithm takes into account the delay in the onset and maximum of calcium events to determine the order of calcium signal propagation and build a dynamic graph, as well as to evaluate a complex of various parameters of calcium activity. The granularity of individual event pairs running sequentially in time allowed us to analyze changes in the network dynamic between different states of cultures by further applying network analysis algorithms. Future studies can rely on this approach to investigate significant changes in the patterns of calcium dynamics at the individual propagation level. The main limitation of the proposed algorithm is the detection of signal transmission based only on the event’s start and finish moments, rather than the detection of calcium propagation through space. Moreover, the identification of adjacent cells is based only on contact between corresponding regions, but not on real connections between cells. This can lead to missing links between cells.

The application of the developed algorithm allowed us to investigate the change in calcium wave propagation in astrocytic and mixed cultures when modeling AD. In order to evaluate the impact of altered astrocytes without artificially introducing β-amyloidosis to neurons in the modeling, we developed an original protocol for cocultivating altered astrocytes with healthy nerve cells. In the pharmacological model of β-amyloidosis, recombinant fibrillar Aβ1-42 was introduced to monoastrocytic cultures for 14 days, after which healthy nerve cells of the hippocampus obtained from mouse embryos (E18) were planted on the altered astrocytes. After replanting healthy cells, no β-amyloid was added in the process of cocultivation. After 14 days of cocultivation, calcium imaging and an analysis of calcium signaling between cells were performed.

The results of the present study demonstrated for the first time that 14 days after completion of the modeling of amyloidopathy, the calcium activity of monoastrocytic cultures does not differ from that of the control group. However, when cocultured, altered astrocytes affected the activity of healthy nerve cells and changed their calcium signaling pattern, leading to an increase in the number of cell-to-cell calcium signal transmissions. There have been no studies of cell-to-cell calcium signal transmission, although there have been studies devoted to the activity of individual astrocytes both in vitro and in vivo. For example, the hyperactivity of astrocytes adjacent to amyloid plaques mediated by activation of metabotropic purinergic receptors has been described [46,47].

The analysis of calcium dynamics in astrocyte cultures obtained from 5xFAD transgenic mice also revealed no changes in cell-to-cell calcium signal transmission. Of particular interest is that when healthy nerve cells were cocultured with 5xFAD astrocytes, a pronounced inhibition of cell-to-cell calcium signal transmission was observed. In contrast, the number of isolated calcium events was maintained.

The study of the secretory profile of astrocytes in the modeling of β-amyloidosis showed that the expression of IL-1β mRNA was increased in altered astrocytes, which indicated the development of sterile neuroinflammation and was consistent with the literature [48,49].

Unexpected for us were data on the increase in the level of GDNF expression by astrocytes after modeling β-amyloidosis, since neurodegenerative processes are traditionally associated with a decrease in the production of neurotrophic factors. However, there is little published data on the role of GDNF in Alzheimer’s disease. In mouse aging models, GDNF levels have been reported to decrease in the cerebral cortex and hippocampus of accelerated-aging mice [50,51]. Data on GDNF in AD are scarce and contradictory. Several studies reported an increase in GDNF levels in the cerebrospinal fluid [52] and plasma [53] of AD patients. On the other hand, in a postmortem study of middle temporal gyrus tissues of patients with AD, GDNF expression was reduced [54]. In [55], serum GDNF levels were significantly reduced in patients with mild cognitive impairment and AD patients. We suggest that the increase in GDNF mRNA expression by astrocytes 14 days after the modeling of chronic β-amyloidosis is an adaptive response aimed at maintaining cell viability.

An important point in understanding the mechanisms of the effect of altered astrocytes on the calcium activity of neuronal cells during cocultivation is the decrease in the expression of NR2B subunit mRNA of the NMDA glutamate receptor. One of the possible neurodegenerative mechanisms in AD is the hyperstimulation of glutamate receptors, mainly NMDA, which leads to an excessive increase in Ca^2+^ levels, causing excitotoxicity and further neuronal death [56]. Moreover, since numerous studies have demonstrated that the Aβ protein oligomer stimulates calcium influx through the NMDA receptor in the pathogenesis of AD, NMDA receptor antagonists are considered potential therapeutic agents for presymptomatic AD [57,58]. Differences in the subunit composition of the NMDAR complex and its subcellular localization provide functional diversity but also determine the role of NMDAR in the induction of neurotoxicity, including that induced by Aβ. It is important to note the role of astrocytes in maintaining the balance of neurotransmitters.

A recent study by Ortiz-Sanz et al. [59] showed that NMDAR NR2B and PSD-95 subunit levels were abnormally elevated in the postsynaptic terminals of the human prefrontal cortex in the early stages of AD, as well as in the hippocampus of 3xTg-AD mice, which was correlated with Aβ42 loading. A study by Marshall [60] is of even greater interest: it showed that a simultaneous increase in extrasynaptic NR2B and a decrease in synaptic NR2B may be responsible for the development of tau pathology and synaptic dysfunction, respectively. Astrocytes can also express NMDA receptors. To date, there are almost no data on the expression of different NMDA subunits in neurodegeneration. However, Li et al. [61] showed that a blockade of astrocytic GluN2A or GluN2B exacerbates Aβ-induced synaptotoxicity, suggesting that astrocytic GluN2A and GluN2B provide synaptoprotection. This was consistent with our data. A decrease in the expression level of the GluN2B subunit in the modeling of β-amyloidosis may explain the induction of changes in healthy neurons during coculturing.

## 4. Materials and Methods

### 4.1. Ethics Statement

All experimental procedures were carried out in accordance with Act 708n (23 082010) of the Russian Federation National Ministry of Public Health, which states the rules of laboratory practice for the care and use of laboratory animals; and Council Directive 2010/63 EU of the European Parliament (22 September 2010) on the protection of animals used for scientific purposes. The animals were kept in the special pathogen-free (SPF) vivarium of the Lobachevsky State University of Nizhny Novgorod. The vivarium had a valid veterinary certificate (no. 52-005921), dated 1 July 2022, for the keeping, breeding, and sale of laboratory animals, and a previous veterinary certificate (no. 52-005777) dated 15 June 2020. The lighting regime of the vivarium was 12/12; the animals had free access to food and water. Newborn C57BL/6 mice (P1–P5) were killed with cervical vertebra dislocation. The study was approved by the Bioethics Committee of Lobachevsky University (protocol no. 42 from 15 October 2020).

### 4.2. Primary Nervous Cultures

The following groups of cultures were used in the experiments:

1. Primary cortical astrocyte cultures without treatments (n = 10);
2. Primary cortical astrocyte cultures with β-amyloid (n = 8);
3. Primary 5xFAD cortical astrocyte cultures (n = 8);
4. Cocultivation of primary astrocyte cultures and hippocampal cultures (neurons and astrocytes) without treatment (n = 6);
5. Cocultivation of β-amyloid primary astrocyte cultures and hippocampal cultures (neurons and astrocytes) (n = 6);
6. Cocultivation of 5xFAD primary astrocyte cultures and hippocampal cultures (neurons and astrocytes) (n = 6).

As a positive control, we used primary cultures of hippocampal cells (n = 10), in which local neural networks capable of spontaneous activity were formed. A detailed description of the assessment of the activity of primary hippocampal cultures and the relevance of the developed algorithm for the study of neuronal cultures is given in Section 2.1.5. (lines 319–380).

As a negative control, we used the MCA-205 cell line (mouse fibrosarcoma) of a nonneuronal genesis. Calcium intercellular signaling is not typical for these cells: there are no signal transduction and network interactions. Therefore, we assumed that the fluctuations in the calcium ion concentration in the cytoplasm of MCA cells were “biological noise” and used them as a negative control.

In total, the calcium activity of 15,121 cells was analyzed.

#### 4.2.1. Isolation of Primary Astrocyte Cultures

The research object was the primary dissociated monoastrocytic cultures of the cerebral cortex of C57BL/6 mice (P1–P5). A detailed description of obtaining monoastrocytic cultures is given in Mitroshina et al. [23]. In brief, after mechanical grinding of the cerebral cortex tissue, the cells were dissociated using a trypsin-EDTA solution (ThermoFisher, Waltham, MA, USA). The nutrient medium for cultivating monoastrocytic cultures was DMEM culture medium (PanEco, Moscow, Russia) supplemented with 10% fetal calf serum (PanEco, Russia), 0.5 mM L-glutamine, 1% sodium pyruvate (ThermoFisher, Waltham, MA, USA), and 2% B27 (ThermoFisher, Waltham, MA, USA). The cells were cultured for 7 days, after which the passage of the cells was carried out. A Versene–trypsin solution (3:1) (PanEco, Moscow, Russia) was used to remove the cells from the substrate.

The initial cell density of monoastrocytic cultures at planting was 4500 cells/mm^2^. The viability of monoastrocytic cultures on 35 DIV was 98.04 ± 0.81%.

Primary astrocyte culture viability was maintained under constant conditions of 35.5 °C, 5% CO_2_, and a humidified atmosphere in a cell-culture incubator (Binder, Tuttlingen, Germany) for more than 30 days [23].

#### 4.2.2. Isolation of Primary Hippocampal Cultures

E18 mouse embryos were used to obtain primary hippocampal cell cultures. Surgical removal of the brain from the cranium and isolation of the hippocampus were performed. The hippocampal tissue was pulverized mechanically and dissociated by treating the hippocampal tissue with 0.25% trypsin (ThermoFisher, Waltham, MA, USA). Neurobasal medium (ThermoFisher, Waltham, MA, USA) supplemented with fetal calf serum, L-glutamine, and nutritional supplement B27 (ThermoFisher, Waltham, MA, USA) was used as a nutrient medium. Cultivation was performed according to a previously developed protocol [32]

The initial cell density of neuronal hippocampal cell cultures was 7000–9000 cells/mm^2^. The viability of hippocampal was 97.55 ± 0.26%.

#### 4.2.3. Obtaining Primary Cultures of Astrocytes from 5xFAD Mice

In our work, we used two protocols developed by us to model β-amyloidosis as a key component of AD. One of them was the culture of astrocytes obtained from 5xFAD mouse embryos as a model of the familial form of AD. The line is characterized by the presence of 5 mutations in *APP and PSEN1* transgenes: *APP* KM670/671NL (Swedish), *APP* I716V (Florida), *APP* V717I (London), *PSEN1* M146L, *PSEN1* L286V; they contribute to the rapid development of amyloidosis [48].

Cultivation of primary cultures of astrocytes obtained from 5xFAD mice was generally performed in accordance with the protocol outlined in Section 4.2.1. After isolation of the cerebral cortex, the cortex of each embryo was placed in a separate Eppendorf in a thermoshaker for the time necessary to perform genotyping at 37 °C and 750 rpm.

During genotyping using PCR, the presence of *PSEN1* and *APP* genes was determined.

The following primers were used:

*PSEN1*-1644—AATAGAGAACGGCAGGAGCA;

*PSEN1*-1645—GCCATGAGGGCACTAATCAT;

*PSEN1/APP/*control-7338—CTAGGCCACAGAATTGAAAGATCT;

*PSEN1/APP/*control-7339—GTAGGTGGAAATTCTAGCATCATCC;

*APP*-3610—AGGACTGACCACTCGACCAG;

*APP*-3611—CGGGGGTCTAGTTCTGCAT.

PCR conditions were as follows: 95 °C for 3 min, 35 cycles of 95 °C for 30 s, 64 °C for 1 min, 72 °C for 1 min, and 72 °C for 2 min on a C1000 Touch thermocycler (Bio-Rad Laboratories, Inc., Hercules, CA, USA).

The result of the PCR was determined with an electropherogram after electrophoresis in agarose gel. Cultures included in the “5xFAD” group were prepared from the cerebral cortex of embryos, in the tissues of which the presence of transgenes was confirmed; wild-type cultures included in the “Control” group were prepared from the cerebral cortex of embryos, in the tissues of which PCR did not confirm the presence of transgenes [62].

#### 4.2.4. Cocultivation of Monoastrocytic and Mixed Neuronal Cultures

After modeling amyloidopathy as a key pathophysiological component of Alzheimer’s disease, healthy hippocampal nerve cells were cocultivated to monoastrocytic cultures at 21 DIV. The material for cocultivation was dissociated cells of the cerebral cortex of C57BL/6 mouse embryos at day 18 of gestation (E18). Isolation of primary hippocampal cultures is described in Section 4.2.2.

### 4.3. β-Amyloidopathy Model Based on Synthetic Aβ42

**Figure 11:**
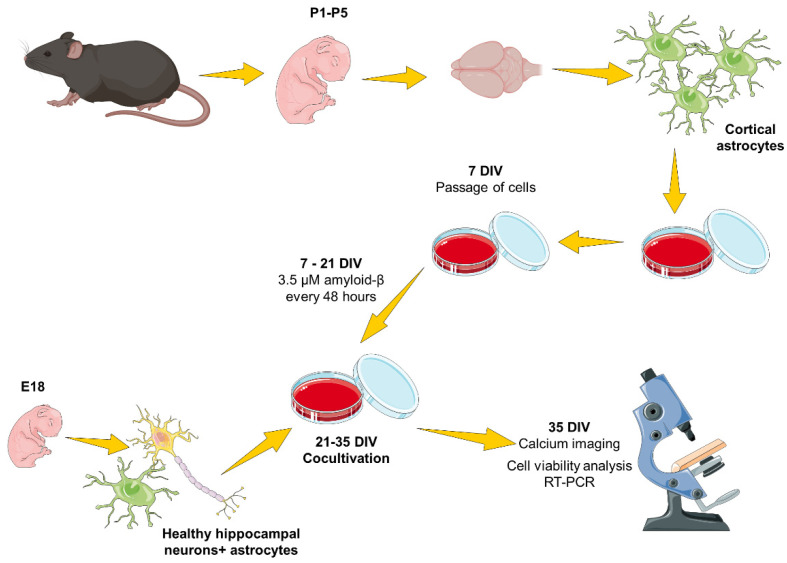
Scheme of experiment. Newborn P1-5 mice were used to obtain the cultures of cortical astrocytes. After euthanasia, the cerebral cortex, subjected to mechanical and enzymatic dissociation, was surgically isolated. The cells were cultured for 7 days in an astrocyte culture medium (see Section 4.2), after which the cells were passaged to remove neurons from the culture completely. After passaging, β-amyloid at a concentration of 3.5 µM was added to astrocytic cultures at each medium change for 14 days to simulate AD. On day 21, dissociated healthy hippocampal cells derived from C57BL/6 mouse E18 embryos were replanted. After that, cultivation continued on the neurobasal medium for culturing neuronal cultures. Cocultivation of AD astrocytes and healthy hippocampal nerve cells lasted for 14 days, after which calcium network activity was recorded using calcium imaging, and cells were harvested for RT-PCR (the figure was created using https://smart.servier.com/ (accessed on 18 August 2022), a publicly available image database).

Aβ (1-42) synthetic peptide (H-1368, Bachem, Bubendorf, Switzerland) was dissolved in 100% 1,1,1,3,3,3 hexafluoro-2-propanol (HFIP) at 6 mg/mL and incubated for 1.5 h until complete dissolution at 37 °C with stirring. After incubation, the protein was dried at room temperature in a laminar flow cabinet. After drying, the protein was dissolved in DMSO (PanEco, Russia) to a concentration of 5 mM. The solution was then brought to a concentration of 100 μM with a 10 mM HCl solution. The obtained Aβ42 was applied to the culture medium at a concentration of 3.5 μM, starting from the first day after culture passaging (DIV 14) for 14 days with each change of the culture medium (Figure 11). A detailed protocol for modeling β-amyloidopathy is given in the paper by Mitroshina et al. [32].

### 4.4. Immunocytochemical Analysis

The following protocol was used to assess the presence and ratio in the culture of Alzheimer astrocytes and healthy nerve cells. At 17 DIV, the cultured astrocytes were transduced with the AAV-CMV-mCherry viral vector to visualize the cultured cells. Next, immunocytochemical staining was performed on DIV 35 after hippocampal cell replantation (Figure 2). Fixation was performed with 4% PFA solution (PanReac & AppliChem USA, Chicago, IL, USA) supplemented with 4% sucrose. Unmasking was carried out with a solution of 0.1% Triton X100 in PBS. For immunocytochemical labeling, the following antibodies were used: chicken anti-GFAP (Cat# Ab4674, Abcam, Cambridge, UK) at a dilution of 1:500 and mouse anti-NeuN (Cat# Ab104224, Abcam, Cambridge, UK) at a dilution of 1:500. The following antibodies were used as secondary antibodies: Alexa Fluor 488 goat anti-mouse (1:1000, Cat# A-11001 ThermoFisher, Waltham, MA, USA) and Alexa Fluor 555 anti-chicken (1:1000, Cat# A-21437 ThermoFisher, Waltham, MA, USA). The exposure time was 2 h for primary antibodies and 1 h for secondary antibodies. Thus, both the original cortical astrocytes and the replanted hippocampal astrocytes were stained with anti-GFAP antibodies.

**Figure 12:**
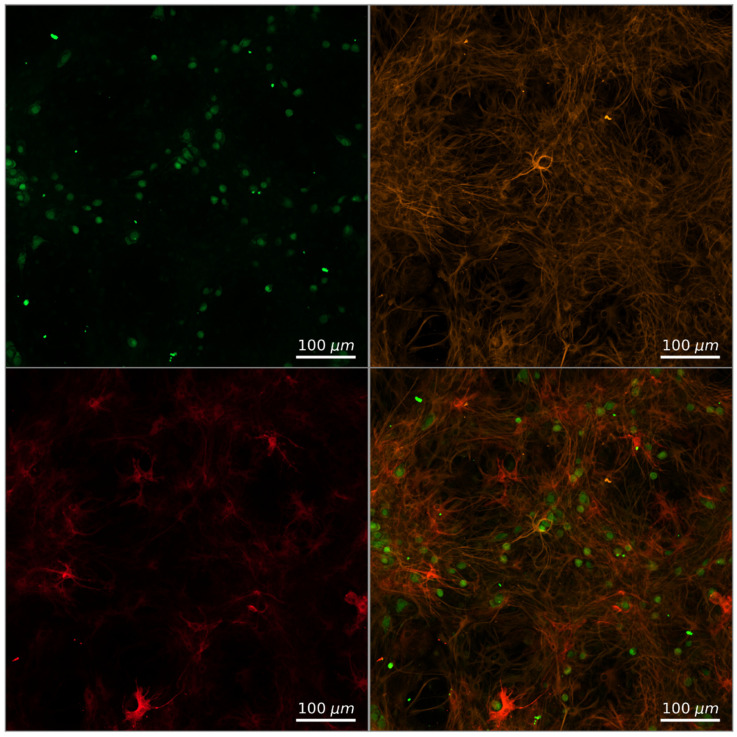
A representative example of immunocytochemical staining of a culture of cocultivated astrocytes treated with AB42 and healthy nerve cells. Green channel—neurons labeled with primary anti-NeuN antibodies and secondary antibodies AlexaFluor 488; orange channel—astrocytes labeled with primary anti-GFAP antibodies and secondary antibodies AlexaFluor 555; red channel—cortical astrocytes transduced with AAV-CMV-mCherry prior to cocultivation. Scale bar: 100 μm. A Plan-Apochromat 10×/0.3 lens was used to acquire the images.

It was shown that after two weeks of cocultivation, the culture contained both the original astrocytes of the cerebral cortex, in which the red fluorescent protein mCherry was visualized, and the astrocytes and hippocampal neurons introduced during cell grafting (Figure 12). The viability of cell cultures after transduction with the AAV-CMV-mCherry vector was 97.62 ± 0.38%. The transfection efficiency was 82.35 ± 4.51%. Representative examples of the morphology of monoastrocitic cultures and cocultivated hippocampal neurons and cortical astrocytes on 35 DIV are shown in Supplementary Figure S2.

### 4.5. Calcium Imaging

A laser scanning microscope LSM 800 (Zeiss, Jena, Germany) with an objective W Plan-Apochromat 10×/0.3 was used for imaging studies of the functional calcium activity of nerve cells. The technique allowed us to visualize the functional architecture of the neuronal network of the culture at the cellular level. Oregon Green 488 BAPTA-1 AM (OGB-1) 0.4 μM (Invitrogen, Carlsbad, CA, USA) was used as a calcium sensor. An LED source with a wavelength of 488 nm was used to excite the fluorescence. A time series of images was recorded to assess the dynamics of intracellular calcium concentration measurements. The resolution of the obtained image was 512 × 512 pixels, the size of the field of view was 693 × 693 µm, and the image recording frequency was 2 Hz.

The obtained imaging data were processed in the Astrocyte Laboratory program (certificate of state registration of the software program 2021612870 dated 25 February 2021 version 1.0) and implemented in the MATLAB environment [23,63]. This was used to perform video filtering, image alignment, and automatic detection of cell boundaries. The developed algorithm for constructing a dynamic graph used data arrays generated by the Astrocyte Laboratory and saved as mat files:

Time series of intensity;

An array describing the area that points belonged to;

Filtered video sequence;

Area adjacency matrix.

In the area adjacency matrix,

(25) $$A=\left(a_{ij}\right),\;a_{ij}=\left\{\begin{array}{c} 1,\;\;\;\;\;if\;i\;and\;j\;cells\;are\;contiguous\;on\;the\;boundary,\\ 0,\;\;\;\;otherwise.\; \end{array}\right.$$

On the data obtained using calcium imaging of MCA-205 “biological noise” cultures, cell areas were identified using a watershed segmentation algorithm. Moving objects (debris, dead cells, etc.) on the frame were excluded from further analysis through visual evaluation. The calcium signal for individual cells was determined as the average value of the fluorescence intensities of the pixels in the area corresponding to the cell. The difference between signal and baseline was taken as the change in cell signal intensity. The resulting set of changes in the intensity of each cell was used to estimate the standard deviation of biological noise. The calculated value multiplied by 3 was used as the biological noise level. Deviations of the signal from the baseline by a value exceeding 3 standard deviations of biological noise were taken as calcium events.

### 4.6. Cell Viability Analysis

To identify the nuclei of dead cells and the total number of cell nuclei in the primary astrocyte cultures, propidium iodide (Sigma-Aldrich, Darmstadt, Germany) and bis-benzimide (Hoechst 33342) (Sigma-Aldrich, Darmstadt, Germany) were used. Solutions of 5 μg/mL propidium iodide and 1 μg/mL bis-benzimide were added separately to the culture medium 30 min before viability registration. The stained cultures were observed using a Leica DMIL HC inverted fluorescence microscope (Leica, Wetzlar, Germany). The proportion of dead cells was calculated as the ratio of nuclei stained with propidium iodide to the total number of nuclei [64].

### 4.7. RT-PCR

Total RNA from primary astrocytic cultures was isolated using ExtractRNA reagent (Evrogen, Moscow, Russia) according to the manufacturer’s protocol. The amount of isolated RNA was determined using UV spectrophotometry (Nanodrop One, ThermoFisher Scientific, Waltham, MA, USA). An MMLV RT kit and random primer (Evrogen, Moscow, Russia) were used for reverse transcription.

*Oaz1*_fw 5′-AAGGACAGTTTTGCAGCTCTCC-3′

*Oaz1*_rv 5′-TCTGTCCTCACGGTTCTTGGG-3′

*Hif1α*_fw1 5′-GCAATTCTCCAAGCCCTCCAAG-3′

*Hif1α*_rv1 5′-TTCATCAGTGGTGGCAGTTGTG-3′

*Bdnf*_fw 5′-CCCAACGAAGAAAACCATAAGGA-3′

*Bdnf*_rv 5′-CCAGCAGAAAGAGTAGAGGAGGCT-3′

*TrkB*_fw 5′-TTTCCGCCACCTTGACTTGTCT-3′

*TrkB*_rv 5′-GTCGGGGCTGGATTTAGTCTCC-3′

*Gdnf*_fw 5′-CCTTCGCGCTGACCAGTGACT-3′

*Gdnf*_rv 5′-GCCGCTTGTTTATCTGGTGACC-3′

*Gfrα1*_fw 5′-TGTCTTTCTGATAATGATTACGGA-3′

*Gfrα1*_rv 5′-CTACGATGTTTCTGCCAATGATA-3′

*Tnf*_fw 5′-GCCCACGTCGTAGCAAACC-3′

*Tnf*_rv 5′-TGGTTGTCTTTGAGATCCATGCC-3′

*Il1b*_fw 5′-GCCCATCCTCTGTGACTCATGG-3′

*Il1b*_rv 5′-GTTCATCTCGGAGCCTGTAGTGC-3′

*Il10_*fw 5′-AAGCATGGCCCAGAAATCAAGG-3′

*Il10*_rv 5′-CAGGGGAGAAATCGATGACAGC-3′

*Bcl-2*_fw 5′-CTACGAGTGGGATGCTGGAGATG-3′

*Bcl-2*_rv 5′-TCAGGCTGGAAGGAGAAGATGC-3′

*Grin2b*_fw 5′-GGTGAGGTGGTCATGAAGAGGGC-3′

*Grin2b*_rv 5′-GGGTTCTGCACAGGTACGGAGTTG-3′

*App*_fw 5′-ATGCAGAATTCCGACATGACTCAGGA-3′

*App*_rv 5′-CACCATGAGTCCAATGATTGCACCTT-3′

*Gja1*_fw 5′-TGCGCTTCTGGGTCCTTCAGAT-3′

*Gja1*_rv 5′-CTGCGCCACTTTGAGCTCCTCT-3′

qPCR conditions: 50 °C for 2 min, 95 °C for 10 min, 40 cycles of 95 °C for 15 s, 60 °C for 60 s on an Applied Biosystems 7500 thermocycler (Applied Biosystems, ThermoFisher Scientific, Waltham, MA, USA) using the reaction mixture qPCRmix-HS SYBR + LowROX (Evrogen, Moscow, Russia).

Data processing was carried out using the ΔΔCt method and a control sample in which the expression level was taken as one. *Oaz1* was used as a housekeeping gene to normalize the obtained data. One of the most important characteristics of a reference gene candidate is a stable expression, i.e., a gene with a small coefficient of variation (CV) and a maximum fold change < 2 (MFC, the ratio of the maximum and minimum values observed within the dataset). In addition, a mean expression level lower than the maximum expression level subtracted by 2 standard deviations (SD) was a prerequisite for a candidate housekeeping gene.

Based on these requirements, we selected the Oaz1 gene with the following parameters: 0.45 (SD), 3.78 (CV, %), and 1.51 (MFC). For a visual comparison, the GAPDH was 0.74 (SD), 5.75 (CV, %), and 6.37 (MFC); these values were several times higher than the maximum allowable requirements for candidates [62].

### 4.8. Statistical Analysis

A Mann–Whitney–Wilcoxon test was performed to compare two unrelated groups that were non-normally distributed (normal distribution was assessed using the Shapiro–Wilk test). To counteract the problem of multiple comparisons, *p*-values were corrected using the Holm–Bonferroni method. A one-way ANOVA was followed by a Bonferroni post-hoc test for multiple comparisons, which was used to analyze normally distributed data. Differences between groups were considered significant when the *p*-value was less than 0.05.

## 5. Conclusions

Our study showed that astrocytes subjected to Alzheimer’s disease modeling can change the functional pattern of the calcium activity of healthy nerve cells during cocultivation. The opposite effects were found in astrocytes treated with AB42 (activation of calcium signaling) and the coculture of healthy cells with 5xFAD astrocytes in mouse embryos (suppression of calcium signaling). In this regard, it seems relevant to further study astrocytic–neuronal interactions as an important factor in the regulation of the functional activity of brain cells during neurodegenerative processes. The approach to the analysis of streaming imaging data developed by the authors makes it possible to describe the signal transmission between network cells and to visualize the functional network architecture in the form of dynamic graphs, which is a promising tool for studying the collective calcium dynamics of nerve cells.

## Data Availability

The algorithms for imaging data processing developed by the authors are stored in the following repositories and are available on request: dynamic graph construction algorithm implemented in MATLAB: https://github.com/TVK-dev/dynamic_graph (accessed on 12 December 2022); script for building illustrations: https://github.com/TVK-dev/paper_dynamic_graph_astro_neuro_ab_fad (accessed on 12 December 2022); algorithm for marking the boundaries of calcium events: https://github.com/TVK-dev/TruEvent (accessed on 12 December 2022).

## Supplementary Materials

The supporting information can be downloaded at: https://www.mdpi.com/article/10.3390/ijms232415928/s1.
