# Supplementary material for: Novel Algorithm of Network Calcium Dynamics Analysis for Studying the Role of Astrocytes in Neuronal Activity in Alzheimer’s Disease Models
Source: Int J Mol Sci. 2022 Dec 14;23(24):15928. doi: 10.3390/ijms232415928 (PMC9781291; doi:10.3390/ijms232415928)
Supplement: Supplementary file 1 [file ijms-23-15928-s001.zip › Supplementary_1.pdf]

Supplementary 1

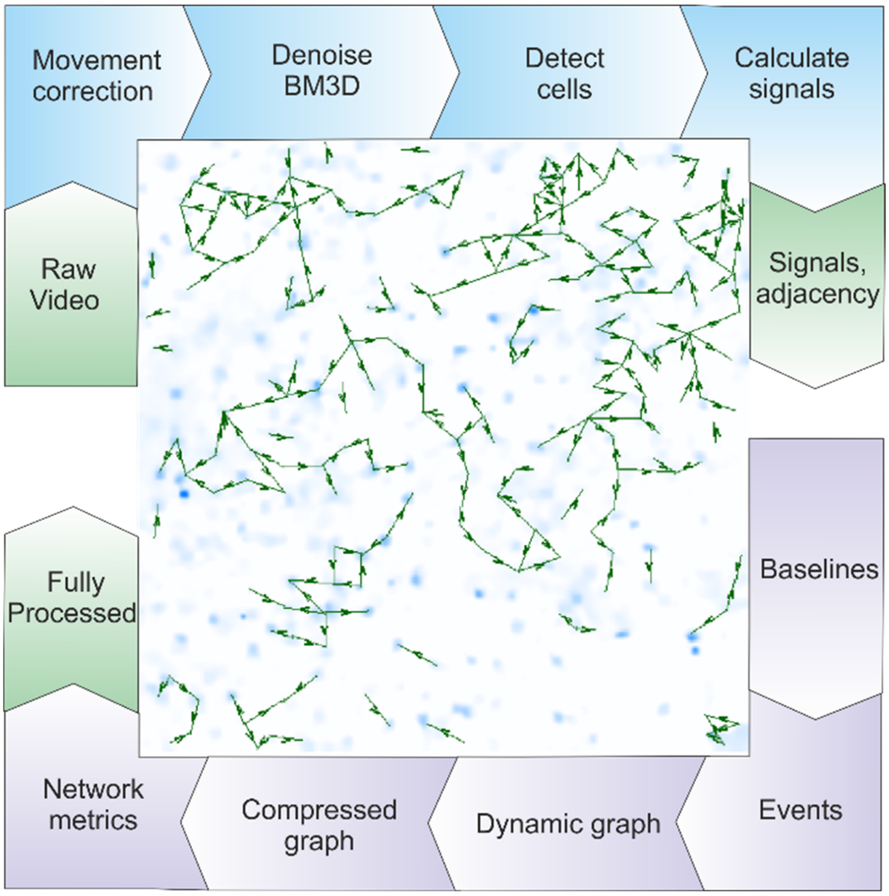

Supplementary Figure S1. The developed algorithm pipeline: the signal preprocessing (upper blue blocks) and event based network reconstruction (bottom purple block).

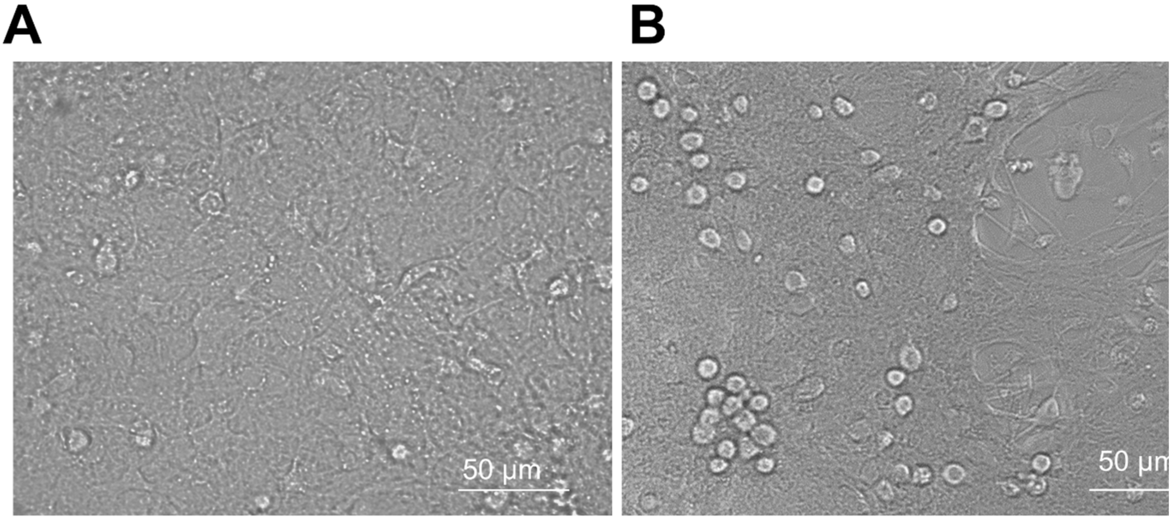

Supplementary figure S2. A - Morphology of primary astrocyte cultures on 35 DIV, representative wide-field light microscopy image. **B -** Morphology of co-cultivated hippocampal neurons and cortical astrocyte on 35 DIV (14 day of cocultivation), representative wide-field light microscopy image. Scale bars – 50 µm.
